# Bilateral simultaneous anterior cruciate ligament tears treated with single staged simultaneous primary repair: A case report

**DOI:** 10.1016/j.ijscr.2022.107670

**Published:** 2022-09-17

**Authors:** Xiuyi A. Yang, Harmen D. Vermeijden, Robert O'Brien, Jelle P. van der List, Gregory S. DiFelice

**Affiliations:** a Orthopaedic Sports Medicine and Trauma Service, Department of Orthopaedic Surgery, Hospital for Special Surgery, NewYork-Presbyterian, Weill Medical College of Cornell University, New York, NY, United States; b Amsterdam UMC, University of Amsterdam, Department of Orthopaedic Surgery, Amsterdam, the Netherlands; c Spaarne Gasthuis Hospital, Department of Orthopaedic Surgery, Hoofddorp, the Netherlands

**Keywords:** Anterior cruciate ligament, Bilateral anterior cruciate ligament injury, Primary repair, Skiing injuries, Case report

## Abstract

**Introduction and importance:**

Simultaneous bilateral anterior cruciate ligament (ACL) injuries are a rare injury pattern within the literature. There is not a consensus optimal management of this injury. Bilateral primary ACL repair in a single stage surgery provides knee stability with a minimally morbid surgery in a single rehabilitation period. This case report offers another option for surgeons to consider in the treatment of this rare injury.

**Case presentation:**

A 45-year-old female skier presented with simultaneous bilateral isolated proximal anterior cruciate ligament injuries. MRI demonstrated bilateral proximal ACL tear patterns which were amenable to primary ACL repair. The patient subsequently underwent acute single-staged arthroscopic primary ACL repair with suture augmentation of both knees. She attained rehabilitation milestones and was fully cleared to return to sporting activities one year post-operatively. Two years post-operatively the patient continues to do well with excellent clinical outcomes.

**Clinical discussion:**

The other treatment modalities reported in the literature were single staged and two staged ACL reconstruction with either autograft or allograft. While single staged procedures are more time and cost efficient, the primary concern is that simultaneous rehabilitation of bilateral ACL reconstructions may lead to severe quadriceps deconditioning. Primary ACL repair poses a potential solution as a minimally morbid surgery with faster rehabilitation from surgery.

**Conclusion:**

Due to the limited invasiveness and morbidity of ACL primary repair with suture augmentation, simultaneous primary repair surgery could be an excellent treatment option for this rare patient population, saving time and cost while providing appropriate knee stability.

**Level of evidence:**

Level IV, Case Report.

## Highlights

• Single-stage bilateral ACL repair is a viable treatment option in a patient with simultaneous bilateral ACL injuries
• ACL repair might be an excellent treatment option for this population due to its minimally morbid nature.
• Simultaneous bilateral ACL repair allows for early return to ambulation and range of motion

## 1 Introduction

Anterior cruciate ligament (ACL) tears are among the most common knee injuries, with an annual incidence of 200,000 tears in the United States. Among patients presenting to clinic with ACL tears, a small subset (2–4 %) present with bilateral knee pathology [1], [2]. Most often, patients do not sustain these injuries simultaneously but during two separate events. Therefore, while ACL reconstruction remains the gold standard treatment of isolated ACL injuries, limited evidence for optimal treatment of simultaneous bilateral ACL injuries is available, with only a few reported cases in the literature [3], [4], [5], [6].

Currently, treatment for bilateral ACL injuries varies from conservative management to surgical reconstruction in either a simultaneous or staged fashion [7], [8], [9]. In recent years, several investigators have revisited primary ACL repair due to the potential advantages of ACL repair over ACL reconstruction [10], [11]. One such advantage is that ACL repair is less invasive than reconstruction because the native ligament is preserved while avoiding donor site morbidity [12]. Furthermore, ACL repair patients have shown an earlier return of range of motion (ROM) when compared to ACL reconstruction patients [13]. These factors promote a quicker achievement of rehabilitation milestones following ACL repair, which is particularly important in complex cases involving bilateral functional impairment [3].

We present the case of a 45-year-old patient with simultaneous bilateral proximal ACL injuries treated with single-stage bilateral knee arthroscopic primary ACL repairs performed by senior author GSD, a fellowship trained orthopaedic surgeon with over 20 years of experience in ligamentous knee surgery. The patient was informed and consented to the publication of the data concerning her case. This case report was performed in line with SCARE 2020 criteria [23].

## 2 Case report

A 45-year-old female with no significant past medical or surgical history presented in 2020 for clinical evaluation three weeks after torsional trauma of both knees during a single ski injury. She came to clinic with moderate bilateral knee pain and recurrent instability of both knees while walking. No medication, family, psychosocial history were notable. Physical examination of the left knee in the clinic showed 130° degrees of flexion with a grade two Lachman, grade two pivot shift test but was otherwise ligamentously stable exam and was neurovascularly intact distally. Although physical examination of the right knee showed similar findings, she had a grade one rather than a grade two pivot shift on examination.

**Fig. 1:**
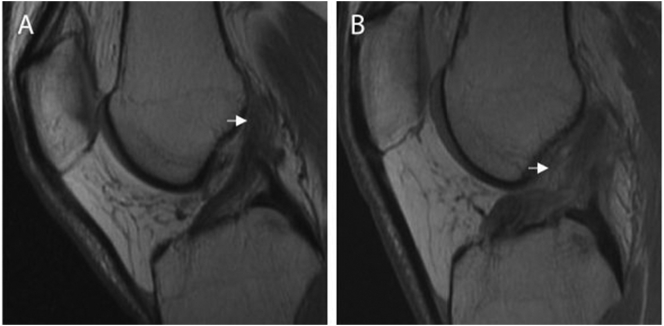
MRI of the same patient with bilateral ACL injuries. (A) Sagital T1 imaging of the right knee shows a proximal avulsion type 1 complete ACL tear (arrowhead). (B) Sagital magnetic resonance image T1 of the left knee shows a proximal type 2 complex complete ACL tear (arrowhead).

Magnetic resonance imaging (MRI) of both knees was obtained and revealed a type 2 proximal ACL tear of the left knee, and a type 1 proximal avulsion ACL tear of the right knee (Fig. 1) [14]. There were no significant concurrent meniscal or chondral injuries. After discussing treatment options which included non-operative management, primary repair and reconstruction with either allograft or autograft, the patient was indicated for a single-staged bilateral knee procedure, in which an intraoperative decision, based on tear type and tissue quality, would determine the ultimate treatment for both injuries as previously described [15].

No pre-operative optimization was required. The patient underwent surgery five weeks after injury. Examination under anesthesia revealed both knees with full ROM, grade two Lachman, grade two pivot shift, and no varus or valgus laxity.

**Fig. 2:**
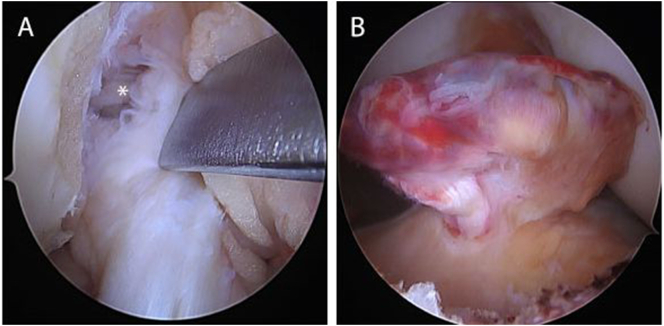
Arthroscopy of the ACL tear in the right and left knee of the same patient. (A) Arthroscopic view of a right knee viewed from the anterolateral portal with the patient supine and the knee in 90° flexion. A proximal avulsion tear with excellent tissue quality is seen. (B) Arthroscopic view of a left knee viewed from the anterolateral portal with the patient supine and the knee in 90° flexion shows a complex tear pattern of the ACL.

**Fig. 3:**
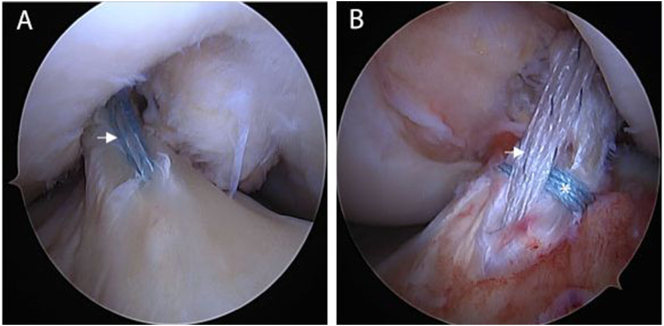
Arthroscopic evaluation after reattachment of bilateral ACLs in the same patient. (A) Arthroscopic view of a right knee viewed from the anterolateral portal with the patient supine and the knee in 90° flexion. Shown is the completed ACL repair with suture augmentation (arrowhead). (B) Arthroscopic view of a left knee viewed from the anterolateral portal with the patient supine and the knee in 90° flexion. Shown is a completed ACL repair with suture augmentation (arrowhead). Note that there is an extra horizontal stitch at the midsubstance of the ligament (asterisk).

Starting with the right knee, arthroscopic examination revealed a proximally torn ACL with excellent tissue quality (Fig. 2A). The length and quality of tissue were deemed eligible for ACL repair. Using a scorpion suture passer (Arthrex, Naples, FL), a locking stitch of #2 FiberWire (Arthrex, Naples, FL) was passed through the anteromedial (AM) bundle first. The posterolateral (PL) bundle was then sutured in the same manner using a #2 TigerWire (Arthrex, Naples, FL). With the knee in 115° of flexion, a hole was tapped at the femoral PL bundle insertion site. After passing the repair stitches from the PL bundle through the eyelet of a 4.75-mm vented BioComposite SwiveLock suture anchor (Arthrex, Naples, FL), this anchor was then deployed at the anatomical PL bundle origin. The same procedure was subsequently repeated for the AM bundle. However, for optimal footprint visualization, this anchor was deployed with the knee in 90° of flexion. In addition, the suture anchor was preloaded with a FiberTape (Arthrex, Naples, FL), acting as an additional suture tape augmentation to protect the repaired ligament during early rehabilitation. The next step was to fixate the FiberTape distally. Therefore, an ACL guide was used to drill a 2.4-mm tunnel through the anteromedial tibial cortex into the anterior third of the ACL tibial insertion. Using a Ninotol wire, the FiberTape was subsequently retrieved through the tibia and then fixated into the anteromedial cortex in near full extension another suture anchor, after which ACL repair with suture augmentation was completed (Fig. 3A).

The left knee was then approached in similar fashion. Arthroscopic inspection, however, demonstrated the majority of the ligament with a type II proximal tear and with some AM bundle fibers scarred to the femoral wall (Fig. 2B). Nevertheless, it was felt that the tissue quality was sufficient and the tear was proximal enough to have sufficient length to be reapproximated to the femoral wall, and thus that this tear was eligible for repair as well. However, a more complex suture pattern with several locking, looping, and transverse components was performed in addition to the suture augmentation used on the contralateral limb (Fig. 3B). After primary repair of this knee was completed, physical examination of both knees revealed full ROM with a negative Lachman test.

Both knees were placed into a knee orthosis locked in extension in the operating room. The patient was allowed to weight bear as tolerated with crutches and started rehabilitation. All follow-up was performed in the clinic. One week post-operatively the patient returned to clinic and demonstrated 80° and 90° of flexion in her left and right knee, respectively, with negative Lachman tests bilaterally. She regained full bilateral ROM within six weeks. After five months, the patient started running without any symptoms of instability, weakness, or pain. At one-year follow-up, she was cleared for all activities. At two-year follow-up she continued to have bilateral negative Lachman tests and had returned to skiing with no difficulties. Objective anterior knee laxity measurements were obtained using the Lachmeter and demonstrated 5.02 mm and 3.78 mm on the left and right knees respectively, while both knees had a normal knee function (grade A) according to the International Knee Documentation Committee (IKDC) Objective Score. Functional outcomes scores showed a Lysholm score of 95, Cincinnati score of 96, IKDC Subjective score of 87.35, Forgotten Joint Score-12 (FJS-12) of 88, and Anterior Cruciate Ligament-Return to Sport after Injury Scale (ACL-RSI) score of 53.33. When inquired about the patient's perspective on the rehabilitation and current status of her knees in clinic at most recent follow-up the patient stated that she feels secure in the stability of her knees and is confident when skiing.

## 3 Discussion

Simultaneous bilateral ACL injuries are extremely rare, with only a few cases described in the orthopaedic literature [3], [4], [5], [6]. As the literature is limited, the optimal treatment approach remains debatable. Currently, all reported cases underwent surgical reconstruction in either a simultaneous or staged fashion.

This case report suggests that single-staged bilateral ACL repairs, when performing surgery in the relatively acute phase, may be a promising approach given the low morbidity, due to the avoidance of tunnel drilling and the usage of grafts. Over the past decade, there has been increasing advocacy for primary repair surgery in the treatment algorithm of proximal ACL tears. However, no studies in the literature were identified assessing outcomes of patients with bilateral ACL repair. When reviewing studies on unilateral repair outcomes, a recent meta-analysis reported that among 69 patients treated with ACL repair with suture augmentation, there was a 6 % failure rate, 0 % reoperation rate, and good to excellent functional outcomes scores [16]. Nevertheless, this meta-analysis also showed a lack of long-term data supporting ACL repair in the literature. Furthermore, it has been suggested that failure rates of this procedure might be higher than those reported in the ACL reconstruction literature, especially in younger patients [17], [18]. Therefore, future studies are required to compare long-term clinical outcomes of this procedure with the surgical gold standard of ACL reconstruction.

Primary ACL repair with suture augmentation might be an excellent treatment in patients with acute simultaneous bilateral proximal ACL injuries, facilitating a more tolerable rehabilitation process, demonstrated by earlier ROM return [13], [16], [19]. Additionally, patients who have undergone ACL repair show less awareness of their operative knee than those with ACL reconstruction [19]. Finally, patients with simultaneous bilateral ACL injuries treated with single staged bilateral ACL reconstruction have shown increased utilization of physical therapy to return to their preinjury activity level compared to unilateral ACL reconstruction [7]. This indicates that the potential benefits in rehabilitation of ACL repair relative to reconstruction may be magnified in this patient population. It is possible that in the case of a failure of repair with conversion to reconstruction that the overall rehabilitation time is most likely similar to a multi-stage reconstruction while affording the patient the opportunity to avoid an arduous recovery from bilateral ACL reconstructions.

In this patient group, both single and multi-stage surgery have been suggested as treatment options. One advantage of single-stage surgery is avoidance of prolonged delay between injury to surgery, which may decrease the risk for additional meniscal and cartilage surface lesions [20]. While single-stage surgery significantly decreases overall direct surgical costs, it may also lead to fewer sick leave days, shorter overall rehabilitation time, and less opioid consumption, which is both time-efficient and cost-effective [7], [21]. However, there are also disadvantages associated with this procedure. First, some patients with bilateral surgery may reduce activity post-operatively to the extent that persistent significant quadriceps weakness develops despite aggressive rehabilitation protocols [22]. Furthermore, there is some concern for increased re-injury risk after single-stage bilateral post-operative functional knee impairment [3]. This concern, however, may be unfounded as multiple studies have shown similar clinical outcomes after single-stage bilateral ACL reconstruction compared to unilateral ACL reconstruction [3], [7], [8], [21].

Additionally, non-operative treatment could be reasonably pursued in patients over 40 years of age. While no studies have been reported comparing non-operative management to either ACL repair or reconstruction in bilateral ACL injury, operative treatment was chosen after discussing all options with the patient due to their desire to return to down-hill skiing.

In conclusion this case shows that single-stage bilateral arthroscopic ACL repair with suture augmentation in a patient with simultaneous bilateral proximal ACL injuries with good to excellent tissue quality is a potentially viable operative option. Orthopaedic surgeons should take away from this case that because of the minimal invasiveness and morbidity of ACL repair surgery, this procedure might be an excellent treatment option for such severely injured patients when the proper criteria of proximal tear and sufficient tissue quality are present.

## Consent

Written informed consent was obtained from the patient for publication of this case report and accompanying images. A copy of the written consent is available for review by the Editor-in-Chief of this journal on request.

## Ethical approval

This study was exempt for ethical approval at the institution at which this work was performed.

## Funding

There was no funding for this study.

## Author contribution

Xiuyi A. Yang: Development of study concept, data collection, data analysis and interpretation, writing the paper

Harmen D. Vermeijden: Data analysis and writing the paper

Robert O'Brien: Writing the paper

Jelle P. van der List: Data analysis and writing the paper

Gregory S. DiFelice: Development of study concept, data collection, data analysis and interpretation, writing the paper

## Guarantor

Gregory S. DiFelice.

## Research registration number

This work was not a First in Man study.

## Provenance and peer review

Not commissioned, externally peer-reviewed.

## Declaration of competing interest

Gregory S. DiFelice declares he is a paid consultant and receives research grants from Arthrex (Naples, FL, USA).
